# Function of *HIF-1α* in Regulation of Antioxidative Stress of *Tribolium castaneum* Under Hypoxia

**DOI:** 10.3390/insects17030343

**Published:** 2026-03-21

**Authors:** Zhichao Wan, Xiao Li, Yun Wang, Shiyuan Miao, Zhiteng Chen, Sufen Cui, Yujie Lu

**Affiliations:** 1 School of Biotechnology, Jiangsu University of Science and Technology, Zhenjiang 212004, China; 18361816569@163.com (Z.W.); 231211803116@stu.just.edu.cn (X.L.); 2 School of Grain Science and Technology, Jiangsu University of Science and Technology, Zhenjiang 212004, China; 232241821412@stu.just.edu.cn (Y.W.); shy.miao@just.edu.cn (S.M.); chenzhiteng@just.edu.cn (Z.C.); luyjlyj71@just.edu.cn (Y.L.)

**Keywords:** *Tribolium castaneum*, *HIF-1α*, hypoxia, RNA interference, antioxidant stress

## Abstract

Hypoxia-inducible factor (HIF) is recognized as a key transcription factor via regulating a variety of molecular responses to hypoxia, although the details are still unclear. In this study, based on bioinformatics analysis, the expression of the *HIF-1α* gene in *T. castaneum* (*TcHIF-1α*) under hypoxic treatments was determined. After *TcHIF-1α* knockdown by injecting dsRNA, larval mortality, the expression levels of oxidative stress-related genes, and enzymatic activities were measured; DNA damage was also evaluated through single cell gel electrophoresis. The result indicated that *TcHIF-1α* is highly conserved in structure. *TcHIF-1α* exhibited distinct temporal patterns, with a peak after 72 h of exposure to 2% O_2_. Following *TcHIF-1α* knockdown, a significant increase in larval mortality (17.44 ± 5.91%) and moderate DNA damage level was found. This might be accompanied by ROS accumulation, lipid peroxidation (LPO), and suppression of antioxidant enzymatic activities. The expression of genes involved in ROS synthesis (e.g., *NOX*) was significantly upregulated, whereas genes responsible for mitigating oxidative stress (e.g., *OGG1*, *XRCC1*, *PARP1*, *SOD1a*) were markedly downregulated. These findings elucidate the critical role of *HIF-1α* in insect hypoxia adaptation by regulating the antioxidative stress, highlighting its potential as a promising target for developing novel pest control strategies.

## Simple Summary

The red flour beetle (*Tribolium castaneum*) is a major pest that damages stored grains worldwide. Low-oxygen conditions (hypoxia) in sealed storage systems represent an environmentally friendly method for controlling this pest. However, beetles are capable of surviving under hypoxic conditions. In this study, we investigated a key gene, *HIF-1α*, which enables the response to hypoxia. We found that *HIF-1α* is structurally conserved across different insect species. When beetle larvae were exposed to severe hypoxia (2% oxygen), expression of *HIF-1α* was increased. To understand its function, we used RNA interference to knockdown *HIF-1α* and observed the response of beetles to hypoxia. Results showed that beetles without *HIF-1α* were more likely to die under hypoxia and suffered serious DNA damage. Genes responsible for repairing oxidative damage were suppressed. These findings showed that *HIF-1α* helps *T. castaneum* survive under hypoxic environments by regulating antioxidant defenses and repairing DNA damage. Targeting *HIF-1α* might lead to more effective pest control strategies for stored grain protection.

## 1. Introduction

Oxygen (O_2_) is essential for the growth and development of most living organisms on Earth, serving as the final electron acceptor in mitochondrial energy production processes [1]. Under hypoxic conditions, excessive reactive oxygen species (ROS) accumulate in mitochondria, disrupting cell division, differentiation, and signal transduction. This leads to cessation of feeding, development, and reproduction in insects, and ultimately death [2]. Modified atmospheres (MAs) based on hypoxia have been recognized as an effective method for controlling insects during stored-product storage [3].

However, insects inhabit a wide range of ecological environments. Some species live in persistently hypoxic habitats, such as high-altitude regions, whereas others occupy environments that may become completely anoxic, including aquatic habitats, decomposing organic matter, or sealed storage containers. Consequently, many stored-product pests have evolved a high capacity for hypoxia adaptation, enabling them to survive under hypoxic or even anoxic conditions for extended periods. Once returned to normoxic environments, these insects can resume normal growth, development, and reproduction [4]. Hypoxia-inducible factor 1 (HIF-1) is a transcription factor that regulates the expression of downstream genes involved in those bioprocesses in insects under hypoxia; related studies on model organisms such as *Drosophila* and *Callosobruchus maculatus* have been reported [5]. HIF-1 is a heterodimeric DNA-binding complex consisting of α and β subunits, and the expression of the β subunit is stable in the nucleus, whereas the α subunit is active, which could be regulated at the post-translational level [6,7]. These studies have investigated the role of *HIF-1α* on regulation behavior and physiology of insects under hypoxia.

Under normoxia, the conserved proline and asparagine residues of HIF-1α are hydroxylated by a family of hydroxylases, then recognized by von Hippel–Lindau (VHL) protein, and then transferred through the ubiquitin–proteasome pathway. Under hypoxia, HIF-α is accumulated and transported to the nucleus, where it interacts with HIF-1β, regulating the expression of downstream genes [7]. Recent studies revealed that HIF-1α mediates adaptive responses to oxidative stress by nuclear translocation and regulation of gene expression [8,9]. HIF-1α in mitochondria (Mito-HIF-1α) can attenuate apoptosis induced by exposure to hypoxia or H_2_O_2_-induced oxidative stress and reduce the production of ROS [10]. *HIF-1α* can activate a series of mechanism to help cells counteract oxidative stress and promote survival [11]. For instance, by shifting metabolism from mitochondrial oxidative phosphorylation to glycolysis, *HIF-1α* fundamentally reduces electron leakage from the mitochondrial electron transport chain, thereby decreasing the source of ROS [12]. HIF-1 plays an important role in cell death in a human retinal pigment epithelium (RPE) cell line under hypoxic stress [13]. When *HIF-1α* is downregulated in vascular endothelial cells, the nrf2/ho-1 signaling pathway is activated, leading to the downregulation of the genes involved in antioxidant enzymes, such as superoxide dismutase 1 (SOD1), nuclear factor erythroid 2-related factor 2 (NRF2), heme oxygenase 1 (HO1), SOD2 (superoxide dismutase 2), and NQO1 (NADPH: quinone oxidoreductase 1) [10,14].

The red flour beetle *Tribolium castaneum* (Herbst) (Coleoptera, tenebrionidae) is one of the most damaging stored-product insect species throughout the world that has also developed remarkable hypoxia adaptive ability. Hypoxia adaptation capacity in *T. castaneum* varies with life stages. Adults are the most tolerant, followed by late-instar larvae and pupae. In contrast, eggs and early-instar larvae are the most sensitive stages. The adults can survive for nearly 15 days under conditions of 2% oxygen [15]. Although various studies have displayed the behavioral and physiological response of *T. castaneum* to hypoxic conditions, details of the function of HIF-1α and its regulation mechanism are still not fully understood [11,16]. Fewer reports have assessed the functions and molecular mechanisms for HIF1-α regulating *T. castaneum* response to oxidative stress under hypoxia.

Based on the established role of HIF-1 in coordinating cellular responses to hypoxia, we hypothesized that *HIF-1α* is a key regulator of hypoxia tolerance in *T. castaneum*. Specifically, we predicted that TcHIF-1α modulates the expression of genes involved in antioxidant defense and DNA repair to reduce oxidative damage under hypoxic stress. To test our hypothesis, we selected mortality as a direct measure of whole-organism tolerance, and quantified antioxidant enzyme activities (e.g., SOD, CAT) and DNA damage (via comet assay) as functional readouts of cellular oxidative status and genotoxicity. If *TcHIF-1α* is essential for hypoxia adaptation, its knockdown would result in increased mortality, elevated oxidative stress (indicated by dysregulated antioxidant enzyme expression), and accumulated DNA damage following hypoxia exposure.

In this paper, the response of *T. castaneum* third-instar larvae to different hypoxic conditions (2%, 5%, 10% O_2_ balanced with N_2_) under 25 °C was examined. The three oxygen concentrations selected in this study were chosen based on their relevance to both practical pest management and ecological conditions. They encompass the critical range used in modified atmosphere storage for stored-product pest control, where levels between 0% and 11.5% O_2_ are commonly employed, levels below 2% are lethal, and levels between 5% and 10% are typically suppressive for longer-term studies of sublethal effects [17]. In addition, 2% O_2_ is consistently identified as a critical threshold for effective control. A previous study found that at 2% O_2_, complete mortality of *T. castaneum* required 3 days for eggs and early larvae, 10 days for old larvae and pupae, and 15 days for adults. This level can therefore be considered “severe” hypoxia. While higher concentrations such as 4% O_2_ showed intermediate effects, 15 days were required to kill all eggs and old larvae, but other life stages survived, suggesting this as a “moderate” level [18]. Based on the bioinformatic analysis and temporal expression analysis, the regulation mechanism of *TcHIF-1α* in antioxidative stress was further investigated. After *TcHIF-1α* knockdown using RNA interference (RNAi), the changes in DNA damage, peroxide levels, antioxidant enzymatic activities, and related mRNA transcription levels were examined. To investigate the effects of HIF-1a on oxidative stress and DNA damage, we selected eight candidate genes for expression analysis by qPCR. These genes were chosen to represent three key aspects: (1) Antioxidant defense: genes encoding superoxide dismutase (SOD1a and SOD1b), catalase (CAT), and glutathione peroxidase (GPX) were selected to assess the ability to scavenge reactive oxygen species (ROS). (2) ROS production: NOX (NADPH oxidase) was selected as a key enzyme responsible for intracellular ROS generation. (3) DNA repair: genes involved in the base excision repair (BER) pathway, including OGG1 (8-oxoguanine DNA glycosylase), PARP1 (poly [ADP-ribose] polymerase 1), and XRCC1 (X-ray repair cross-complementing protein 1), were selected to evaluate the capacity for repairing oxidative DNA damage. The findings will broaden our knowledge about *HIF-1α* in regulating insect adaptive responses to hypoxia, providing potential targets for developing new pest management strategies, and promoting the efficiency of MAs on stored-product insect control in future.

## 2. Materials and Methods

### 2.1. Insect Rearing and Hypoxic Treatment

*T. castaneum* were reared on a diet with whole wheat flour and dry yeast (19:1 *w*/*w*) in a rearing room at 30 ± 2 °C, 65 ± 5% R.H.

The hypoxia exposure system was adopted from a prior study [19]. Thirty third-instar larvae were placed in 500 mL conical flasks, each sealed with a glass stopper fitted with tubing. The flasks were then connected in series to a gas cylinder via the tubing. Gas mixtures containing 2%, 5%, or 10% O_2_ (balanced with N_2_) were flushed into the flasks. The oxygen concentration of the exhaust gas was monitored using a head-space analyzer (Danensor-checkpoint3, MOCON, Shanghai, China). Once the target concentration was reached, the gas flow rate was minimized to maintain a constant oxygen level. All treatments were carried out in the rearing room to keep the temperature constant. The controls were set in normoxia condition. The experiment was replicated three times (*n* = 30 per replicate). The number of dead larvae was recorded every 48 h until all the larvae died within 27 days. Each flask with 30 insects was used for one experimental unit. Survival data were analyzed using the Kaplan–Meier method. For each oxygen concentration (2%, 5%, 10% O_2_) and the normoxic control (21% O_2_), the survival probability was estimated as a function of time. Individuals that remained alive at the end of the observation period (day 27) were treated as censored observations.

### 2.2. Sequence Analysis and Phylogenetic Tree Construction

The coding sequence of TcHIF-1α was initially identified from the genomic sequence using the NCBI ORF Finder (https://www.ncbi.nlm.nih.gov/orffinder/, accessed on 12 March 2025) with the standard genetic code. The deduced full-length amino acid sequence (GenBank accession no. XP_015835862.1) was then used as a query for conserved domain analysis (Table S1). The isoelectric point of proteins was predicted using Compute pI/Mw (http://web.expasy.org/compute_pi/, accessed on 12 March 2025). The sequence of cDNA and its encoding amino acid sequences was analyzed using DNAMAN7. Multiple sequence alignments were performed using Clustal Omega software (http://www.ebi.ac.uk/Tools/msa/clustalo/, accessed on 13 March 2025). Alignment was performed using MEGA v.11 and GeneDoc v.2.7.0. The structural domain of TcHIF-1α was predicted using SMART (http://smart.emblheidelberg.de/, accessed on 13 March 2025). A neighbor-joining phylogenetic tree was constructed with MEGA v.11 (1000 bootstrap replicates). The GenBank accession numbers of the HIF-1α protein sequences used to perform the sequence alignment are presented in Table S2.

### 2.3. Total RNA Extraction and RT-qPCR

Thirty of the *T. castaneum* third-instar larvae were respectively treated under hypoxia (2% O_2_ + 98% N_2_, 5% O_2_ + 95% N_2_, 10% O_2_ + 90% N_2_) for different lengths of time (6, 12, 24, 48, 72, 96 h), as described above. The experiment was replicated three times. For each biological replicate, three technical replicates were included. After treatment, the surviving insects were collected, frozen in liquid nitrogen, and stored at −80 °C for subsequent experiments.

Total RNA was extracted using TRIzol reagent (Sangon Biotech, Shanghai, China), followed by genomic DNA removal with 10× g DNA Remover Mix (CWBio, Beijing, China). cDNA was synthesized, and reverse transcription was performed using HiFiScript RT MasterMix (CWBio, Beijing, China). The cDNA obtained was stored at −80 °C. Quantitative real-time PCR assays were conducted with MagicSYBR Mixture (CWBio, Beijing, China) on a QuantStudio 3 Real-Time PCR System (Thermo Fisher Scientific, Shanghai, China). For each biological replicate, three technical replicates were included. The data were analyzed using the 2^−ΔΔCt^ method and normalized using *β-actin* as a housekeeping gene [20]. All experiments were performed with three independent replicates. The primer sequences employed in this study are provided in Table S3.

### 2.4. RNA Interference of TcHIF-1α

For RNA interference (RNAi), double-stranded RNA (dsRNA) targeting Tc*HIF-1α* and a negative control dsRNA targeting GFP were synthesized. A 326-bp fragment corresponding to nucleotides 355–680 of the TcHIF-1α coding sequence (GenBank accession no: XP_015835862.1) was selected as the target. The target sequence is provided in Table S4. Primers containing the T7 promoter sequence (5’-TAATACGACTCACTATAGGG-3’) at the 5’ end were designed using Primer 6.0 software.

PCR amplification was performed using *T. castaneum* cDNA as a template, and the purified PCR products were used for in vitro transcription. dsRNA was synthesized using the TranscriptAid T7 High Yield Transcription Kit (Thermo Fisher Scientific, Shanghai, China), according to the manufacturer’s protocol. The synthesized dsRNA was purified, and its concentration and purity were assessed using a NanoDrop 2000 spectrophotometer (Thermo Fisher Scientific, Shanghai, China). The final dsRNA concentration was adjusted to 20 μM for microinjection. To minimize off-target effects, the selected dsRNA sequence was subjected to BLAST analysis (https://blast.ncbi.nlm.nih.gov/Blast.cgi, accessed on 1 March 2026) against the *T. castaneum* transcriptome (NCBI RefSeq database, https://www.ncbi.nlm.nih.gov/refseq/, accessed on 1 March 2026), and no significant homology (E-value < 0.01) to other genes was detected.

For microinjection, 200 nL of *dsHIF-1α* (20 μM) was injected into the body cavity of third-instar larvae between the third and fourth abdominal segments using a Nanoject III microinjector (Drummond Scientific, Broomall, PA, USA). Control larvae were injected with the same amount of *dsGFP*. RNase-free water was the buffer for dsRNA. After injection, larvae were allowed to recover under normoxic conditions for 12 h. Then, 30 live larvae from each group were transferred to hypoxic conditions (2% O_2_ + 98% N_2_) for 72 h. Following exposure, larvae were collected, immediately frozen in liquid nitrogen, and stored at −80 °C for subsequent analyses. qPCR was performed to quantify HIF-1α gene expression and confirm the interference efficiency. The experiment was performed with three independent biological replicates, each consisting of 30 larvae per treatment group.

### 2.5. DNA Damage Detection Using Alkaline SCG Assay

DNA damage under hypoxia was tested using alkaline SCG assay according to a previous study, with slight modifications [21]. The entire experiment was conducted in the rearing room. Briefly, third-instar larvae were randomly divided into four groups (*n* = 30 per group), then subjected to 2% O_2_, ds*HIF-1α* + 2% O_2_, or ds*GFP* + 2% O_2_ treatment. And the insects continuously grown in normoxia served as control. The experiment was replicated three times. Ten surviving larvae from each replicate were collected and rinsed with ice-cold phosphate-buffered saline (PBS, pH 7.4). The samples were transferred to a 1.5 mL microcentrifuge tube containing 200 μL of ice-cold PBS and gently minced with dissecting scissors for 2 min to release cells. The resulting cell suspension was filtered through a 40 μm cell strainer to remove debris and then allowed to settle for 10 min on ice. The supernatant was carefully removed, and the cell pellet was resuspended in 100 μL of PBS.

A total of 20 μL of the cell suspension was mixed with 180 μL of pre-warmed (37 °C) low-melting-point agarose (LMPA, provided in the kit). An aliquot of 80 μL of this mixture was immediately spread onto a microscope slide pre-coated with normal melting-point agarose. A coverslip was placed on the gel, and the slide was kept at 4 °C for 10 min to solidify. After removing the coverslip, slides were immersed in pre-chilled lysis solution (2.5 M NaCl, 100 mM EDTA, 10 mM Tris-HCl, 1% Triton X-100, pH 10.0) and incubated at 4 °C for 1 h in the dark. Following lysis, slides were transferred to an electrophoresis chamber filled with fresh alkaline electrophoresis buffer (300 mM NaOH, 1 mM EDTA, pH >13) and left for 20 min to allow DNA unwinding. Electrophoresis was then performed at 25 V for 20 min at 4 °C in the dark.

After electrophoresis, slides were gently rinsed three times with neutralization buffer (0.4 M Tris-HCl, pH 7.5) for 5 min each, dehydrated in absolute ethanol for 10 min, and air dried at room temperature. Slides were stained with 20 μL of DAPI staining solution (provided in the kit) and covered with a coverslip. For each sample, 50 randomly selected cells (25 from each of two replicate slides) were examined under an Axio fluorescence microscope (Carl Zeiss, Oberkochen, Germany) equipped with an excitation filter (524 nm) and a barrier filter (605 nm). Images were captured and analyzed using Comet Assay Software Project (CASP, http://casplab.com, accessed on 18 July 2025). The percentage of tail DNA (% tail DNA) was used as the primary parameter for DNA damage quantification.

The % of tail DNA (defined as the product of the percentage of DNA in the comet tail and the tail length) was used as an indicator of DNA damage. The percentage of DNA damage was calculated using the following Formula (1).

(1) x =A × 100 ÷ B

where x is the tail DNA (%), A is the tail DNA intensity, and B is the cell DNA intensity.

### 2.6. Oxidative Stress Response Analysis

Thirty third-instar *T. castaneum* larvae were injected with 200 nL ds*HIF-1α*, then transferred to hypoxia and maintained for 72 h. Subsequently, the insects were collected, homogenized in 0.9% NaCl solution (1:9, *w*/*v*), and centrifuged at 2500 rpm, 4 °C for 10 min. The supernatant was collected for the following experiments. Controls were set with ds*GFP*.

The total protein content, ROS level, lipid peroxidation (LPO) content, and antioxidant enzyme (CAT, SOD, POD and GST) activity were assessed according to the manufacturer’s instructions (Nanjing Jiancheng Biotechnology Research Institute, Nanjing, China).

The total protein concentration was determined with a total protein assay kit (Cat# A045-3-2). Briefly, 20 μL of supernatant was mixed with 250 μL of working solution (Reagent One and Reagent Two were combined in a 50:1 volume ratio) and incubated at 37 °C for 30 min. Then, 750 μL of Reagent Three was added. Following a 5 min incubation at room temperature, absorbance was determined at 562 nm. A blank control was prepared using distilled water. Reagent Four was used as the standard solution. The calculation formula is as follows:

(2) $$x=\frac{\left(A_{T}-A_{0}\right)}{\left(A_{S}-A_{0}\right)}\times C_{S}\times N$$

where x is the total protein concentration (μg/mL), A_T_ is the absorbance of the treatment group. A_S_ is the absorbance of the standard solution. A_0_ is the absorbance of the blank. C_S_ is the concentration of the standard solution. N is the dilution factor of the sample.

The ROS was determined with the Reactive oxygen species Assay Kit (Cat# E004-1-1), in which 20 μL of supernatant was mixed with 200 μL of 20µM DCFH-DA and incubated at 37 °C for 60 min. After centrifugation at 1000 g for 10 min, the supernatant was removed, and the cell pellet was collected. Fluorescence intensity was determined with a microplate reader set to excitation/emission wavelengths of 488 nm and 525 nm, respectively. ROS levels were measured as relative quantification.

LPO was determined with a Lipid peroxidation assay kit (Cat# E004-1-1), in which 200 μL of supernatant was mixed with 650 μL of Reagent One working solution (stock solution and diluent were combined in a 3:1 volume ratio) and 150 μL of Reagent Two. The mixture was then incubated at 45 °C for 60 min. Then, the mixture was centrifuged at 4000 rpm for 10 min, and the supernatant was collected for absorbance measurement at 486 nm. A blank control was prepared using distilled water. Reagent Four was used as the standard solution. The calculation formula is as follows:

(3) $$x=\frac{\left(A_{T}-A_{0}\right)}{\left(A_{S}-A_{0}\right)}\times C_{S}÷C_{pr}$$

where x is LPO concentration (U/mg protein), A_T_ is the absorbance of the treatment group. A_S_ is the absorbance of the standard solution. A_0_ is the absorbance of the blank. C_S_ is the concentration of the standard solution. C_pr_ is the total protein concentration.

CAT was determined with a Catalase assay kit (Visible light) (Cat# A007-1-1), in which 100 μL of supernatant was mixed with 1000 μL of Reagent One and 100 μL of Reagent Two. Incubated the mixture at 37 °C for 1 min. Then, 1000 μL of Reagent Three and 100 μL of Reagent Four were added. Absorbance was determined at 405 nm. In the control group, the sample addition step was modified to occur after the addition of Reagent Four. The calculation formula is as follows:

(4) $$x=\left(A_{c}-A_{T}\right)\times 271÷0.1÷60÷C_{pr}$$

where x is CAT concentration (U/mg protein), A_T_ is the absorbance of the treatment group. A_c_ is the absorbance of the control group. C_pr_ is the total protein concentration.

SOD was determined with a Superoxide Dismutase (SOD) assay kit (Cat# A001-3-2,), in which 20 μL of supernatant was mixed with 20 μL of working solution (Reagent Three and Reagent Four were combined in a 1:10 volume ratio) and 200 μL of substrate working solution (Reagent Two and Reagent One were combined in a 1:200 volume ratio). Following a 20 min incubation at 37 °C, absorbance was determined at 450 nm. A control group was prepared using distilled water. For the two groups (sample and control group), 20 µL of enzyme working solution was substituted with 20 µL of enzyme diluent as the blank control. The calculation formula is as follows:

(5) $$x=Y\times 0.5\times \frac{0.24}{0.02}\times N÷C_{pr}$$

(6) $$Y=\left(1-\frac{\left(A_{T}-A_{TO}\right)}{\left(A_{C}-A_{CO}\right)}\right)\times 100\%$$

where x is SOD concentration (U/mg protein), Y is the SOD inhibition rate (%). A_T_ is the absorbance of the treatment group. A_T0_ is the absorbance of the blank of the treatment group, A_c_ is the absorbance of the control group. A_C0_ is the absorbance of the blank of the control group. C_pr_ is the total protein concentration. N is the sample dilution factor.

GST was determined with a Glutathione S—transferase (GSH-ST) assay kit (Colorimetric method) (Cat# A004-1-1), in which 100 μL of supernatant was mixed with 300 μL of substrate solution and incubated in a 37 °C water bath for 30 min. After adding 2 mL of Reagent Two, the sample was centrifuged at 4000 rpm for 10 min. Then, 2 mL of the supernatant was combined with 2 mL of Reagent Three and 0.5 mL of Reagent Four. Following a 15 min incubation at room temperature, the OD value was determined at 412 nm. A blank control was prepared using working solution (Reagent Six and ddH_2_O were combined in a 3:1 volume ratio). A 1 mM GSH solution (Reagent Five and ddH_2_O were combined in a 9:1 volume ratio) was used as a standard solution. The calculation formula is as follows:

(7) $$x=\frac{\left(A_{T}-A_{0}\right)}{\left(A_{S}-A_{0}\right)}\times C_{S}\times 6÷30÷\left(\frac{0.1}{C_{pr}}\right)$$

where x is GST concentration (U/mg protein), A_T_ is the absorbance of the treatment group. A_S_ is the absorbance of the standard solution. A_0_ is the absorbance of the blank. C_S_ is the concentration of the standard solution. C_pr_ is the total protein concentration.

### 2.7. Data Analysis

All statistical analyses were performed using GraphPad Prism 10 (GraphPad Software, San Diego, CA, USA). Data is presented as mean ± standard error of the mean (SEM) unless otherwise stated. The specific statistical tests applied to each experimental endpoint were as follows:

Survival probability: differences in survival curves among treatment groups were assessed by the log-rank.

Two-group comparisons (e.g., ds*HIF-1α* vs. ds*GFP* at a single time point): an unpaired two-tailed Student’s *t*-test was used. For comparisons involving multiple groups (e.g., time-course expression under different oxygen concentrations), one-way ANOVA followed by Dunnett’s post hoc test was applied to compare each treatment group against the control.

Antioxidant enzyme activities (SOD, CAT, GPX) and content of ROS, LPO: Differences between treatment groups were analyzed using one-way ANOVA followed by Tukey’s HSD post hoc test for multiple comparisons. For comparisons involving only two groups (e.g., *dsHIF-1α* vs. *dsGFP* under hypoxia), an unpaired two-tailed Student’s *t*-test was used.

Comet assay (DNA damage): the percentage of tail DNA (% tail DNA) was compared among groups using one-way ANOVA followed by Tukey’s HSD post hoc test.

## 3. Results

### 3.1. Structural and Phylogenetic Conservation of TcHIF-1α

The full-length *TcHIF-1α* gene (GenBank accession no. XP_015835862.1) comprises 3958 bp and contains an open reading frame (ORF) encoding a protein of 895 amino acids, with a predicted molecular weight of 87.4 kDa and a theoretical isoelectric point (pI) of 5.71.

**Figure 1:**
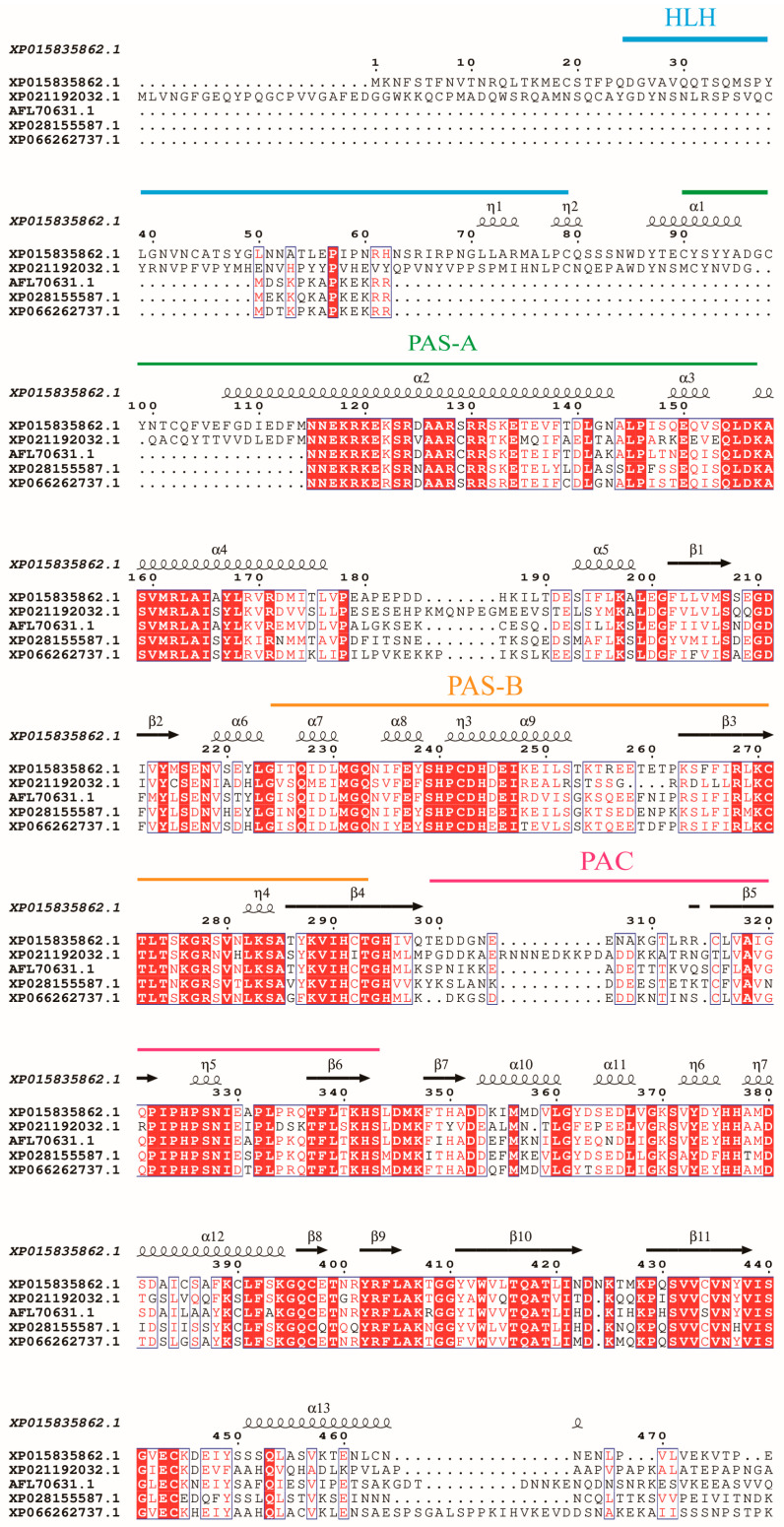
Sequence alignment of HIF-1α. Full-length amino acid sequences from *T. castaneum*(XP_015835862.1), *Helicoverpa armigera* (AMH87782.1), *Callosobruchus maculatus* (AFL_70631.1), *Diabrotica virgifera virgifera* (XP_028155587.1), and *Euwallacea similis* (XP_066262737.1) are aligned by ClustalX 2.1 and edited by ESPript 3.2. Domains of HIF-1αs are marked on the alignment. The α-helix and β-sheet structures were indicated. The helix-loop-helix (HLH) domain, Per-Arnt-Sim (PAS) domain, and PAS-associated C-terminal (PAC) regions are marked with lines above the sequence. Strictly identical residues are highlighted in white letters with a red background. Residues with similar physico-chemical properties are shown in red letters. Alignment positions are framed in blue if the corresponding residues are identical or similar.

Alignment of the sequence with HIF-1α from multiple insect species (Figure 1) showed the following sequence identities: 50.17% with *Helicoverpa armigera* (AMH_87782.1), 62.46% with *Diabrotica virgifera virgifera* (XP_028155588.1), 59.93% with *Euwallacea similis* (XP_066142399.1), and 63.23% with *Callosobruchus maculatus* (AFL_70631.1)**.**

It contains the conserved functional regions: a helix-loop-helix (HLH) domain (residues 24–79 aa) involved in DNA binding, two Per-Arnt-Sim (PAS) domains (PAS-A,90–156 aa; PAS-B,224–293 aa) critical for oxygen sensing, and a C-terminal PAC motif (299–342 aa) stabilizing PAS domain interactions. An alignment of *TcHIF-1α* with homologs from other coleopterans was shown in Table S1.

**Figure 2:**
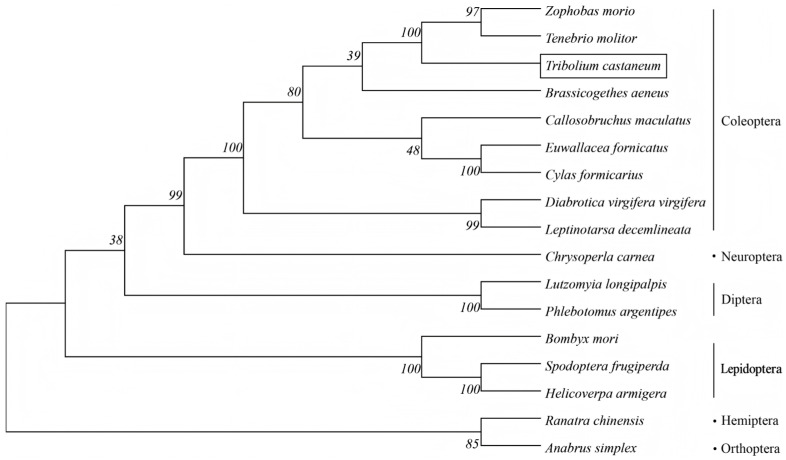
Phylogenetic analysis of HIF1α amino acid sequences from insects with different orders.

Furthermore, the phylogenetic tree of the TcHIF-1α was constructed together with HIF-1α from 16 insect species of Diptera, Hymenoptera, Lepidoptera, Coleoptera, Orthoptera, and Hemiptera using maximum-likelihood phylogenetic analysis. There was strong bootstrap support for the division of TcHIF-1α homologs into one major phylogenetic branch with five distinct clusters, each containing several HIF-1α homologs from different species. TcHIF-1α showed about 68.17% average sequence identities with other Coleopteran HIF-1α (Figure 2, Table S1).

TcHIF-1α exhibited the highest amino acid sequence identity with *Zophobas morio* (Coleoptera) (86.69%), which was relatively close to species in Lepidoptera, Neuroptera, and Diptera insects (with an average identity of 49.72%), and showed greater divergence from Hemiptera and Orthoptera (the average identity was 48.66%).

### 3.2. Survival Probability of T. castaneum Larvae Under Different Hypoxic Conditions

**Figure 3:**
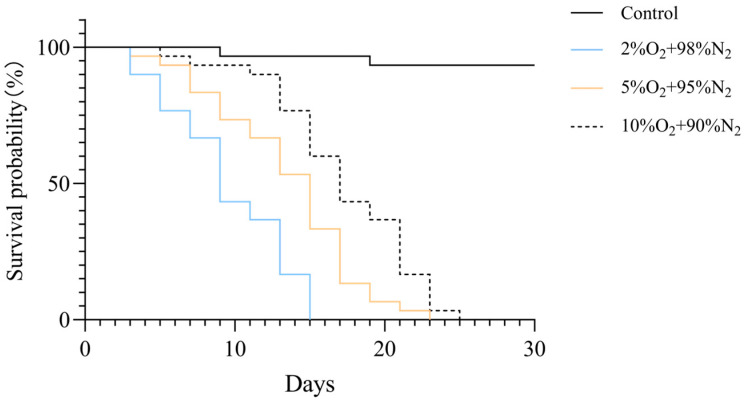
Kaplan–Meier survival curves of *T. castaneum* third-instar larvae exposed to different oxygen concentrations. The overall difference among groups was assessed by the log-rank test (*p* < 0.001).

To assess the impact of oxygen concentration on larval survival, Kaplan–Meier survival curves were generated for each treatment group (Figure 3). Exposure to reduced oxygen concentrations significantly decreased survival of *T. castaneum* larvae (log-rank test, *p* < 0.001). Median survival times were 9 days for 2% O_2_, 15 days for 5% O_2_, 17 days for 10% O_2_, and over 27 days for normoxic controls.

### 3.3. Temporal Expression of TcHIF-1α

**Figure 4:**
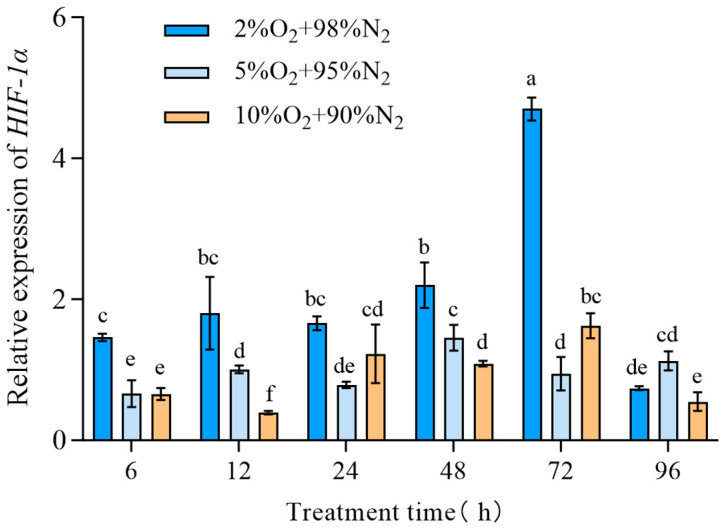
The expression of *TcHIF-1α* under different hypoxic concentrations. Data are expressed as mean ± SE (*n* = 3 independent biological replicates, each consisting of 30 larvae). Values followed by different letters were significantly different by one-way ANOVA with Tukey’s test (*p* < 0.05). Different letters indicate significant differences among groups.

The expression of *TcHIF-1α* in *T. castaneum* third-instar larvae under different hypoxic conditions (2% O_2_ + 98% N_2_, 5% O_2_ + 95% N_2_,10% O_2_ + 90% N_2_) was examined and found to be dependent on both exposure time and oxygen concentration. Generally, the relative expression of *TcHIF-1α* was highest in insects treated with 2% O_2_ for 72 h, and lowest in those treated with 10% O_2_ for 12 h (Figure 4).

### 3.4. Effect of HIF-1α on Survival of T. castaneum Larvae Under Hypoxia

**Figure 5:**
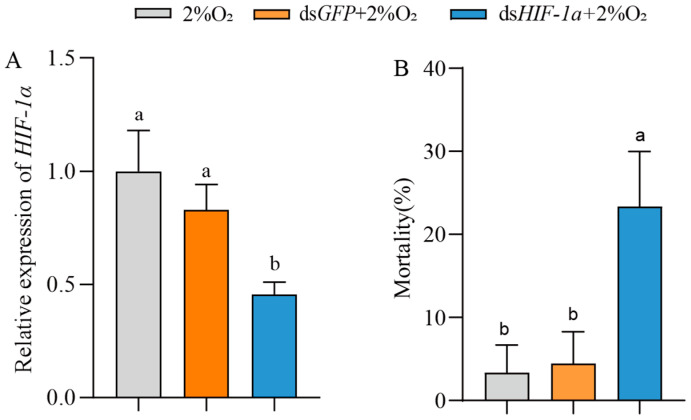
The effect of *HIF-1α* on the mortality of *T. castaneum* third-instar larvae under hypoxia_._ (**A**) Expression of *TcHIF-1α*, and (**B**) mortality of third-instar larvae treated with 2% O_2_, *dsGFP* + 2% O_2_ and *dsHIF-1α* + 2% O_2_, respectively. Data are expressed as mean ± SE (*n* = 3 independent biological replicates, each consisting of 30 larvae). Values followed by different letters were significantly different by one-way ANOVA with Tukey’s test (*p* < 0.05).

To examine the role of *HIF-1α* in insect survival under hypoxia, *T. castaneum* third-instar larvae were injected with ds*HIF-1α*, then treated with 2% O_2_ for 72 h. The controls were set with ds*GFP* + 2% O_2_ and only 2% O_2_. Compared with the controls, the expression of *TcHIF-1α* in insects treated with *dsHIF-1α* + 2% O_2_ was significantly downregulated (54.30 ± 3.86%) (Figure 5A). Correspondingly, the mortality of insects treated with ds*HIF-1α* + 2% O_2_ was significantly increased (Figure 5B). There was no significant difference between the two controls.

### 3.5. DNA Damage of T. castaneum Larvae After HIF-1α Knockdown Under Hypoxia

**Figure 6:**
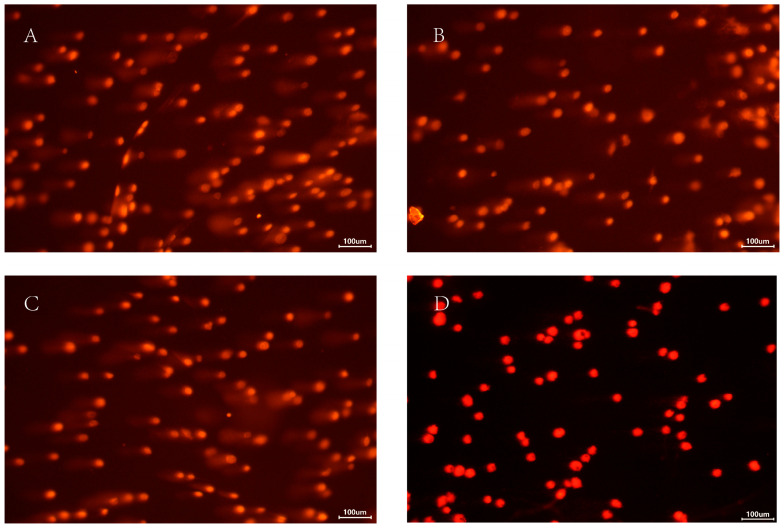
The comet analysis of *T. castaneum* third-instar larvae at different treatments. (**A**) ds*HIF-1α* +2% O_2_ group, (**B**) ds*GFP* +2% O_2_ group, (**C**) 2% O_2_ group, and (**D**) Normoxia.

Larvae were treated with *dsHIF-1α* under 2% O_2_, *dsGFP* under 2% O_2_, or 2% O_2_ only. The effect of HIF-1α on DNA damage regulation in *T. castaneum* third-instar larvae was then examined using the Single Cell Gel Electrophoresis (SCGE) assay. The controls were set with normoxia (Table 1, Figure 6). Based on the value of % tail DNA, the DNA damage in cells is classified into the following five grades: Grade 0 (<5%—no damage), Grade 1 (5% to 20%—mild damage), Grade 2 (20% to 40%—moderate damage), Grade 3 (40% to 95%—high damage), and Grade 4 (>95%—severe damage).

Among all groups, larvae treated with dsHIF-1α under 2% O_2_ sustained the most extensive DNA damage. Based on our damage assessment criteria, this level was categorized as moderate (Figure 6). Correspondingly, the value of % tail DNA in this group was much higher than in other groups; the value of % tail DNA in different groups was *dsHIF-1α* +2% O_2_ > *dsGFP* + 2% O_2_ > 2% O_2_ > normoxia (Table 1). Although there was a significant difference between the ds*GFP* + 2% O_2_ and the 2% O_2_ group, the DNA damage of both groups belonged to Grade 1 (mild damage). The DNA damage in normoxia belonged to Grade 0 (no damage).

### 3.6. Effect of HIF-1α on Antioxidative Stress of T. castaneum Larvae Under Hypoxia

**Figure 7:**
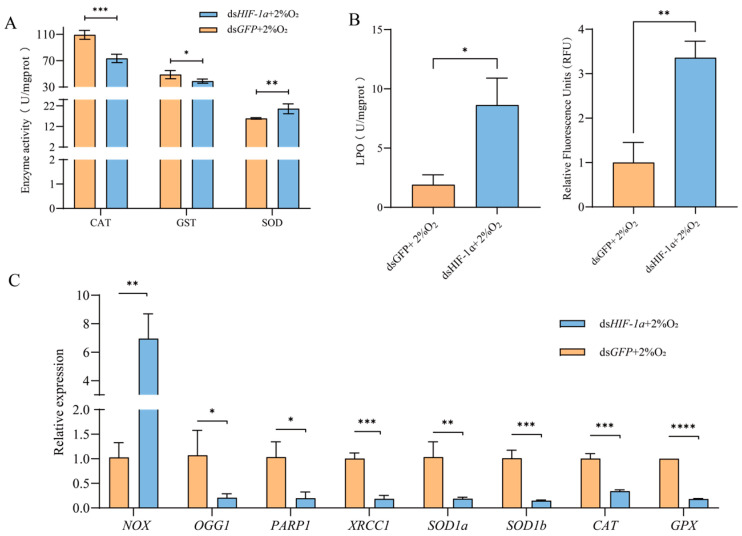
Changes in antioxidative stress of *T. castaneum* third-instar larvae after *HIF1α* knockdown under hypoxia. (**A**) Enzyme activity, (**B**) peroxide content, and (**C**) gene expression. CAT, catalase; GST, glutathione S-transferases; SOD, superoxide dismutase; LPO, lipid peroxide; ROS, reactive oxygen species. *NOX,* NADPH oxidases; *OGG1,* 8-oxoguanine DNA glycosylase-1; *XRCC1,* X-ray repair cross complementing 1; *PARP1,* poly (ADP-ribose) polymerase 1; *SOD1a*, superoxide dismutase 1a, *SOD1b*, superoxide dismutase *1b*; *CAT,* catalase; *GPX,* glutathione peroxidase. Data are expressed as mean ± SE (*n* = 3 independent biological replicates, each consisting of 30 larvae). The significant differences are denoted with * *p* < 0.05, ** *p* < 0.01, *** *p* < 0.001, **** *p* < 0.0001 using an independent *t*-test.

After *HIF-1α* knockdown, the changes in antioxidative stress of *T. castaneum* third-instar larvae under hypoxia, including the activities of antioxidant enzymes (CAT, SOD, and GST), contents of ROS and LPO, and related genes’ expression, were examined. And the control was treated with ds*GFP* (Figure 7).

Compared with the control, the activities of CAT and GST in insects after *TcHIF-1α* knockdown were significantly inhibited, while SOD activity was increased (Figure 7A). The levels of both LPO and ROS were significantly higher in the ds*HIF-1α* group than in the ds*GFP* group. (Figure 7B,C).

Following *TcHIF-1α* knockdown, the expression levels of eight selected genes were significantly altered. Among these, seven genes—*OGG1*, *PARP1*, *XRCC1*, *SOD1a*, *SOD1b*, *CAT*, and *GPX*—were significantly downregulated, whereas NOX expression was upregulated compared with the control (Figure 7C).

## 4. Discussion

The quality and production of grain are severely compromised by pests. Therefore, pest control has been a major focus in grain storage [2]. In recent years, controlled atmosphere storage techniques, such as hypoxic sealing, have been widely adopted in grain storage. However, the gradual evolution of hypoxic adaptability in pests has brought a challenge to the application of this eco-friendly grain storage technology [3]. Research has shown that *TcHIF-1α* plays a critical role in insect adaptation to hypoxia. While most studies have focused on the roles of *HIF-1α* in biological behaviors, metabolic adjustments, and tissue remodeling, research into its regulatory mechanisms of stored-product insect adaptation to hypoxia remains limited [2,4,6]. This study aimed to investigate the function and molecular regulatory mechanism of *HIF-1α* through examining the expression of *HIF-1α* under different hypoxic conditions, DNA damage, and changes in antioxidative stress of *T. castaneum* third-instar larvae after *HIF-1α* knockdown using RNAi. The study might provide theoretical guidance for enhancing the efficacy of MAs in pest control.

As previously reported, the TcHIF-1α subunit shared high similarity and domain structure with homologs from *Helicoverpa armigera*, *Callosobruchus maculatus*, *Diabrotica virgifera virgifera*, and *Euwallacea similis*. TcHIF-1α belongs to the bHLH-PAS family of transcription factors, based on the presence of basic helix-loop-helix (bHLH) and PAS domains. Conserved domains such as bHLH, PAS, and PAC were found. Among these domains, the HLH domain facilitates DNA binding, while the PAS-A and PAS-B domains mediate oxygen-dependent protein interactions. HLH-PAS domains in HIF-1α function as a transcription factor and coactivator for transcriptional regulation of biological responses to environmental and intracellular changes [22]. The C-terminal PAC motif stabilizes these interactions, as observed in *C. elegans* homologs [23]. And the PAC domain, which connects with the PAS domain, acts as a trans-activation domain at the C-terminus of HIF-1α [22]. The sequence alignment further showed that TcHIF-1α was highly conserved in Lepidoptera and Coleopteran. The high homology of amino acid sequence between *T. castaneum* and other Coleopteran, especially *Zophobas morio*, was also observed (Figure 2 and Table S2).

Hypoxia exposure significantly affected the survival of *T. castaneum* larvae in a concentration-dependent manner. Kaplan–Meier survival analysis revealed that median survival times were 9 days under 2% O_2_, 15 days under 5% O_2_, and 17 days under 10% O_2_ (log-rank test, *p* < 0.001). Normoxic controls maintained >98% survival throughout the 27-day observation period, with median survival not reached. These results demonstrate that even under severe hypoxia (2% O_2_), larvae were able to survive for approximately 1 week, indicating a considerable adaptive capacity to low-oxygen conditions. The observed concentration-dependent reduction in survival is consistent with previous studies on stored-product insects exposed to hypoxia. For example, exposure of *T. castaneum* adults to 2% O_2_ for 10 days resulted in approximately 90% mortality, and nearly 100% mortality after 15 days, while exposure to 4% O_2_ required 15 days to reach 90% mortality [24].

Under hypoxic conditions, the transcriptional expression of *TcHIF-1α* exhibited temporal dynamic changes, with higher expression levels observed at lower oxygen concentrations during the treatment. Similarly, *HIF-1α* showed temporal dynamic changes in transcriptional expression under hypoxia in the gut of *Aedes aegypti* [25]. Following *TcHIF-1α* knockdown, DNA damage in *T. castaneum* larvae under hypoxic conditions was markedly more severe, displaying typical DNA fragmentation (20% tail DNA). The olive tail moment was greater in insects treated with ds*HIF-1α* +2%O_2_ than the controls (ds*GFP* + 2%O_2_, only 2%O_2_, and normoxia). It has been reported that the DNA of *T. castaneum* last-instar larvae treated with hypoxia (2% O_2_ + 98% N_2_) for 6 days were significantly damaged due to the accumulated ROS and decreased electrons under hypoxia [21]. In this study, ROS levels were also significantly increased following *TcHIF-1α* knockdown. Furthermore, the accumulation of LPO may further promote ROS production, thereby aggravating damage to DNA and proteins [26].

Correspondingly, the activities of three typical antioxidant enzymes (CAT, SOD, and GST) were found to be significantly changed after *TcHIF-1α* knockdown. The activities of CAT and GST were decreased, while SOD activity was increased. In addition, the expressions of related genes (*CAT*, *GPx, SOD1a*, *SOD1b, NOX, OGG1, PARP1*, and *XRCC1*) were also significantly changed. It has been reported that the nuclear factor kappa B (NF-κB) pathway is activated in vivo following HIF-1α knockdown. Additionally, in Wistar rats fed with AlCl_3_, NF-κB pathway activation was accompanied by decreased activities of CAT and GST [27]. These results suggest that *HIF-1α* may play an important role in regulating the activities of these antioxidative enzymes by activating the expression of their corresponding genes. We also found that the expression of related genes (*CAT* and *GPx*) was downregulated following *TcHIF-1α* knockdown. Previous studies have reported the role of *HIF-1α* in antioxidative stress, yet its detailed mechanisms remain incompletely understood.

*NOX* is a key inducer of cellular superoxide anion (primarily O_2_•^−^) accumulation. Inhibition of *NOX* by a NOX inhibitor significantly reduced superoxide anion levels in rat vascular smooth muscle cells [28]. Superoxide dismutase (SOD), positioned at the forefront of the antioxidant system, catalyzes the dismutation of superoxide radicals (O_2_^−^) [29]. Consequently, a positive correlation may exist between changes in SOD activity and *NOX* expression. Specifically, upregulation of *NOX* expression is likely accompanied by an increase in SOD activity—a relationship supported by our findings (Figure 7). Following *TcHIF-1α* knockdown, the upregulation of *NOX* might result in a marked increase in superoxide-derived products (LPO and ROS) in *T. castaneum* under hypoxia. In response to the elevated O_2_•^−^ levels, SOD activity was upregulated via HIF-independent regulatory pathways (e.g., JNK) in *T. castaneum* [30]. *HIF-1α* can suppress NF-κB signaling by competing for p300 binding. Accordingly, knockdown of HIF-1α in human fetal osteoblasts activates the NF-κB pathway and upregulates *NOX* expression [31]. Similarly, activated NF-κB upregulates *NOX* expression in *Drosophila* [32]. Consistent with these reports, our study in *T. castaneum* also demonstrates that *NOX* expression is upregulated following *HIF-1α* knockdown, further supporting the role of *HIF-1α* in regulating *NOX* expression (Figure 7C).

*SOD1a* and *SOD1b*, two paralogs of the human *SOD1* gene, are highly homologous (>70%) and crucial for preventing oxidative damage via eliminating superoxide anions (O_2_^•−^) [33]. In this study, the transcriptional levels of *SOD1a* and *SOD1b* were downregulated following *TcHIF-1α* knockdown. Previous studies have reported that *SOD1* expression is significantly modulated upon *HIF-1α* upregulation in murine lung cancer cells [34]. These results suggest that *SOD1* expression may be regulated by *HIF-1α*, although the underlying mechanism remains unclear. *OGG1*, a housekeeping gene widely expressed in eukaryotic cells, recognizes and excises the 8-oxoG–an (8-oxo-7,8-dihydroguanine) oxidized lesion in double-stranded DNA by activating the base excision repair (BER) pathway, thereby restoring DNA integrity [35]. In the present study, *OGG1* expression was downregulated following *TcHIF-1α* knockdown, which likely contributed to increased DNA damage and ultimately insect mortality (Figure 7). *HIF-1α* and *OGG1* exhibit functional convergence in regulating downstream biological processes, such as the Vascular Endothelial Growth Factor (VEGF) pathway. When *OGG1* expression was downregulated via siRNA, the stability of the HIF-1α protein was attenuated in pulmonary artery endothelial cells under hypoxic conditions. [36]. Downregulation of *TcOGG1* may further reduce HIF-1α protein stability, thereby functionally deactivating the HIF-1α protein. PARP1 acts as a sensor of DNA strand breaks, while XRCC1 serves as a scaffold protein; they could recruit additional factors to efficiently repair oxidative-stress-induced DNA single-strand breaks [37,38]. PARP1 has been demonstrated to be a hypoxic response regulator and is modulated by *HIF-1α* [39]. In this study, downregulation of *XRCC1* and *PARP1* following *TcHIF-1α* knockdown likely compromised DNA repair capacity in insect cells under hypoxia, and the details need to be further studied in future.

## 5. Conclusions

This study investigated the important role of *HIF-1α* in regulating the antioxidative stress response of *T. castaneum* larvae under hypoxic conditions. Using RNAi technology to knock down *TcHIF-1α*, we systematically examined the responses of third-instar larvae to hypoxia, including mortality, DNA damage, antioxidant enzyme activities, and related gene expression. The results indicate that knockdown of TcHIF-1α was associated with increased mortality, accelerated DNA damage, and altered activities of key antioxidant enzymes (GST, CAT, SOD), as well as changes in the expression of their corresponding genes. These findings might enhance our understanding of insect adaptation to hypoxia and may provide a potential target for improving the efficacy of modified atmospheres in stored-product pest control.

## Figures and Tables

**Table 1:** DNA damage in *T. castaneum* third-instar larvae at different treatments using Single Cell Gel Electrophoresis (SCGE) assay. Data are expressed as mean ± SE (*n* = 3 independent biological replicates, each consisting of 30 larvae). Different letters above the bars indicate significant differences among groups (one-way ANOVA with Tukey’s HSD test, *p* < 0.05).

	%TailDNA	Tail Moment	Olive Tail Moment
ds*HIF-1α* +2% O_2_	20.56 ± 2.30 a	15.61 ± 3.45 a	10.04 ± 3.31 a
ds*GFP* +2% O_2_	11.23 ± 2.19 b	3.39 ± 1.31 b	3.29 ± 2.66 b
2% O_2_	9.14 ± 2.85 bc	3.20 ± 1.07 b	4.11 ± 1.72 b
normoxia	4.37 ± 0.92 c	1.68 ± 0.83 c	1.05 ± 0.93 c

## Data Availability

The original contributions presented in this study are included in the article/Supplementary Material. Further inquiries can be directed to the corresponding author.

## Supplementary Materials

The following supporting information can be downloaded at: https://www.mdpi.com/article/10.3390/insects17030343/s1, Table S1: GenBank accession numbers of HIF-1α protein used in the alignment. Table S2: Identity of amino acid sequence of TcHIF with HIF from representative animal species. Table S3: Primers used for RT-qPCR and dsRNA synthesis. Table S4: Target sequence for *dsHIF-1α*.
