# Supplementary material for: Function of HIF-1α in Regulation of Antioxidative Stress of Tribolium castaneum Under Hypoxia
Source: Insects. 2026 Mar 21;17(3):343. doi: 10.3390/insects17030343 (PMC13027032; doi:10.3390/insects17030343)
Supplement: Supplementary file 1 [file insects-17-00343-s001.zip › insects-4148750-supplementary.pdf]

Function of *HIF-1α* in regulation of antioxidative stress of *Tribolium castaneum* under hypoxia

Zhichao Wan[2], Xiaoli[2], Yun Wang[1], Shiyuan Miao[1], Zhiteng Chen[1], Sufen Cui[1*] ,Yujie Lu[1]

Affiliation 1; Sufen18@just.edu.cn
Affiliation 2;

* [1]School of Grain Science and Technology, Jiangsu University of Science and Technology, Zhenjiang 212004, China; [2]School of Biotechnology, Jiangsu University of Science and Technology, Zhenjiang 212004, China.

Z.W and X.L contributed equally to this paper

Table S1 GenBank accession numbers of HIF-1α protein used in the alignment
Table S2 Identity of amino acid sequence of TcHIF with HIF from representative animal species
Table S3 Primers used for RT-qPCR and dsRNA synthesis
Table S4 Target sequence for *dsHIF-1α*:

Table S1 GenBank accession numbers of HIF-1α protein used in the alignment

Gene	specie	Accession number	E-value	Identity (%)
HIF-1α	Helicoverpa armigera	AMH87782.1	4e-174	50.17%
	Callosobruchus maculatus	AFL70631.1	0	62.46%
	Diabrotica virgifera virgifera	XP028155588.1	0	59.93%
	Euwallacea similis	XP066142399.1	0.0	63.23%

Table S2.Identity of amino acid sequence of TcHIF with HIF from representative animal species

Order	Species	Gene	Amino acid count	Accession number	Identity
Coleoptera	Tribolium castaneum	TcHIF-1.	895	XP_015835862.1	100%
	Zophobas morio	ZmHIF-1.	802	XP_063918499.1	86.69%
	Tenebrio molitor	TmHIF-1.	802	KAJ3637776.1	85.95%
	Brassicogethes aeneus	BaHIF-1.	844	CAH0550233.1	67.54%
	Leptinotarsa decemlineata	LdHIF-1.	830	AKG92758.1	59.45%
	Diabrotica virgifera virgifera	DvHIF-1.	820	XP028155588.1	59.93%
	Euwallacea similis	EfHIF-1.	970	XP066142399.1	63.23%
	Cylas formicarius	CfHIF-1.	883	XP_060536115.1	60.09%
	Callosobruchus maculatus	CmHIF-1.	833	AFL70631.1	62.46%
Lepidoptera	Helicoverpa armigera	HaHIF-1.	857	AMH87782.1	50.17%
	Spodoptera frugiperda	SfHIF-1.	860	XP_050551329.1	53.35%
	Bombyx mori	BmHIF-1.	673	XP_062532151.1	48.90%
Orthoptera	Anabrus simplex	AsHIF-1.	976	XP_066996422.2	46.00%
Diptera	Lutzomyia longipalpis	LlHIF-1.	1031	XP_055678318.1	49.45%
	Phlebotomus argentipes	PaHIF-1	1087	XP_059610479.1	47.33%
Neuroptera	Chrysoperla carnea	CcHIF-1.	1065	XP_044731135.1	49.16%

Hemiptera	Ranatra chinensis	RcHIF-1.	563	KAL1110206.1	51.31%

Table S3 Primers used for RT-qPCR and dsRNA synthesis

Name	Forward(5' to 3')	Reverse(5' to 3')
qHIF-1α	ATTCAAAGCTCCCGCTGTCT	AATTGGCAGAGCGTTACCCA
qβ-actin	TCCATCATGAAGTGCGATGT	CCACATCTGTTGGAATGTCG
qNox	ACTGGCTTGAAGACTAGAACTAATGC	CTCTAATAATCCTGGCGAGTTGTGG
qOGG1	ATACCTATCGACACGCATGTTTACC	TGTGCCCAACCTGCCAGAG
qPARP1	GATTGTTGCTGCTGTGTGATGTTG	TGCTTTAGGGTCAGGCTCAGTC
qXRCC1	CAAGCAAACGAAAGCACGAATCC	TTGTATGCCGCTAATGACCAAGG
qSOD1a	GCACGATCTGGGGAAAGGGA	TTTCCGGTCCTAGAAATGGC
qSOD1b	CCTCCACATTCACCAGTCGG	TCCACACTGCCATCTTCCTC
qCAT	CAATCGCAACCCGGAGAACT	TTGCGCACTTTGAAGGGACA
qGPx	TCGTAAATGTGGCCTCCCAG	GGTGTGCATCTTTGCCGTTC
dsHIF-1α	GTCAGCGAGTATCTTGGAA	GAAGGATGCGGAATAGGTT
dsGFP	GGATACGCACTCGTCGATTT	TCAACAACTCGGCTCGAATACC

Table S4 Target sequence for *dsHIF-1α*:

Gene	Length	Sequence
HIF-1α	326	GTCAGCGAGTATCTTGGAATTACACAGATTGATCTG<br>ATGGGTCAGAATATTTTTGAGTACAGTCATCCGTGC<br>GATCATGACGAAATCAAGGAAATTTTATCAACAAAG<br>ACCCGGGAAGAAACAGAGACGCCTAAGTCGTTTTT<br>TATCAGGCTTAAGTGTACTCTTACAAGCAAAGGCCG<br>ATCGGTCAATCTCAAATCAGCGACTTACAAGGTCAT<br>CCATTGCACTGGTCACATAGTGCAAACTGAAGATGA<br>CGGAAATGAAGAAAATGCGAAAGGAACTTTGCGCC<br>GATGTCTTGTGGCAATAGGACAACCTATTCCGCATC<br>CTT
